# Supplementary material for: An anionic two dimensional covalent organic framework from tetratopic borate centres pillared by lithium ions
Source: Nat Commun. 2024 Aug 15;15:7031. doi: 10.1038/s41467-024-50953-2 (PMC11327249; doi:10.1038/s41467-024-50953-2)
Supplement: Supplementary file 1 — Supplementary Information [file 41467_2024_50953_MOESM1_ESM.pdf]

An anionic two dimensional covalent organic framework from tetratopic borate centres pillared by interlayer lithium ions

Darosch Asgari[1], Julia Grüneberg[1], Yunkai Luo[2], Hüseyin Küçükkeçeci[1], Samrat Ghosh[1,3], Veniamin Chevelkov[4], Sabrina Fischer-Lang[5], Jérôme Roeser[1], Adam Lange[4], Bruce Dunn[2], Michael Gradzielski[1], Arne Thomas[1*]

[1]Department of Chemistry, Technische Universität Berlin, Berlin 10623, Germany.[2]Department of Materials Science and Engineering, University of California, Los Angeles, California 90095, United States. [3]Inorganic and Physical Chemistry Laboratory, Council of Scientific and Industrial Research (CSIR), Central Leather Research Institute (CLRI), Chennai 600020, India. [4]Research Unit Molecular Biophysics, Leibniz-Forschungsinstitut für Molekulare Pharmakologie, Berlin 13125, Germany. Organic Materials Chemistry, Osnabrueck University, Osnabrueck 49069, Germany.

Table of Contents

1. Synthetic Procedures 3
2. Solid State Nuclear Magnetic Resonance 4
3. PXRD Data and Background 5
4. Calculation of Interpenetration Degree 5
5. Simulation and Refinement Data 6
6. Stability of BPB-COF 7
7. Thermogravimetric Analysis (TGA) 8
8. Sorption Measurements 9
9. BETSI Analysis 10
10. Transmission Electron Microscopy (TEM) 12
11. Scanning Electron Microscopy (SEM) 15
12. Atomic Force Microscopy (AFM) 17
13. Electron Impedance Spectroscopy (EIS) 18
14. ICP-OES Measurements 18
15. Theoretical Simulations of Chemical Shielding 19
16. Reflex Summary Report for Rietveld Refinement 20
17. Supplementary References 23

1. Synthetic Procedures

Lithium tetraphenoxyborate (TPB): An oven dried Schlenk tube equipped with stirrer and septum was charged with phenol (0.753 g, 8.00 mmol, 4.00 eq.), evacuated, and backfilled with Argon for a total of three times. Phenol was dissolved by the addition of anhydr. 1,4-dioxane (30 ml). The reaction was started by the slow addition of LiBH4 (2 M in anhydr. THF, 1 ml, 2 mmol, 1.0 eq.). The reaction mixture was stirred for 30 min at room temperature and then heated to 70 °C and stirred overnight. After cooling to room temperature, the white solid was collected via Schlenk filtration and washed with anhydr. dioxane (10 ml), anhydr. acetone (10 ml) and anhydr. pentane (10 ml). The solid was dried under reduced pressure at 40 °C overnight. TPB was obtained as a white solid (92%, 0.720 g, 1.85 mmol).

^{11}B-NMR (64 MHz, CDCN): δ = 2.52 ppm.

^{11}B-NMR (CP-MAS): δ = 1.77 ppm.

^{7}Li-NMR (78 MHz, CDCN): δ = –1.53 ppm.

^{1}H-NMR (400 MHz, CDCN): δ = 6.65 (m_c, 4H, H-4), 6.95–7.12 (m, 16H, H-2/H-3) ppm.

^{13}C-NMR (100 MHz, CDCN): δ = 119.3 (C-4), 120.0 (C-2)*, 129.5 (C-3)*, 158.6 (C-1) ppm.

HRMS (APCI) (m/z): [M$^-$] calcd for for $C_{24}H_{20}BO_4^-$, 383.1460; found, 383.1451.

IR (ATR): $\tilde{\nu}$/cm-1 = 3037 (w), 1592 (s), 1488 (s), 1437 (w), 1227 (s), 1155 (w), 1094 (w), 1026 (m), 1008 (m), 957 (s), 878 (m), 770 (w), 744 (s), 715 (w), 691 (s), 668 (w).

Biphenol linked borate network (BPB-Poly): An oven dried Schlenk tube equipped with stirrer and septum was charged with 4,4’-biphenol (745 mg, 4.00 mmol, 2.00 eq.), evacuated, backfilled with argon for a total of three times and dissolved in anhydr. THF (30 ml). The reaction was started by the addition of LiBH4 (2.00 M in anhydr. THF, 1 ml, 2 mmol, 1.00 eq.). The colorless suspension was stirred for 6 hours at room temperature under argon atmosphere. The colorless solid was collected through filtration under argon atmosphere, washed with anhydr. THF (4 × 10 ml), and dried under reduced pressure overnight at 100 °C. The polymer was obtained as off-white solid (93%, 695 mg).

Procedure for the synthesis of 4,4’-biphenol borate-COFs (BPB-COF): An oven dried Schlenk tube equipped with stirrer and septum was charged with 4.4’-biphenol (745 mg, 4 mmol, 2.00 eq.), evacuated and backfilled with argon a total of three times and dissolved in anhydr. THF (30 ml). The reaction was started by the addition of $LiBH_4$ (2 M in anhydr. THF, 1 ml, 2 mmol, 1.00 eq.) and stirred for 6 hours at room temperature. Under argon atmosphere the suspension was transferred into a PTFE-lined steel autoclave. After the addition triethylamine (192 mg, 1.9 mmol, 0.265 ml, 1 eq.) and stirring by hand the autoclave was sealed and placed into a pre-heated oven at 150 °C for 5 days. After cooling down to room temperature inside the oven, the solid was collected under

argon atmosphere by filtration and washed with anhydr. THF (4 × 10 ml). The solid was dried overnight under reduced pressure at 100 °C overnight. BPB-COF was obtained as a colorless solid (652 mg, 85%).

Duration	***Triethylamine***	***Crystallinity***	***Surface Area (BET)***
0 days	0 Eq.	Amorphous	4.6 m^2g^{-1}
2 days	1 Eq.	Low	808 m^2g^{-1}
5 days	1 Eq.	Moderate	786 m^2g^{-1}
5 days	0 Eq.	Amorphous	-
12 days	1 Eq.	High	825 m^2g^{-1}

Supplementary Table 1:Overview of the durations for solvothermal treatment and influence of triethylamine with corresponding surface areas.

Exfoliation of BPB-COF Nanosheets

For the exfoliation 1 mg of **BPB-COF**, were suspended in 10 ml of dry methanol under inert atmosphere. The Suspension was sonicated for 30 minutes followed by centrifugation at 10280 g for 10 minutes leaving the nanosheets natant in the solution.

2. Solid State Nuclear Magnetic Resonance

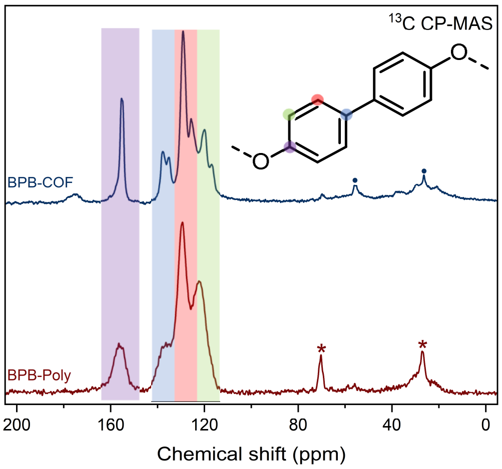

*Supplementary Fig. 1: ^{13}C CP-MAS NMR spectrum of **BPB-Poly** and **BPB-COF** (12 days) and 4,4'-biphenol linker. • represent spinning sidebands and * represents residual THF.*

3. PXRD Data and Background

PXRD samples were prepared under inert atmosphere using plastic specimen holders that can be covered with Kapton foil. This results in additional background. The specimen holder was measured with no sample and the obtained PXRD pattern used as background. Both the background data and sample data were first normalized before subtraction.

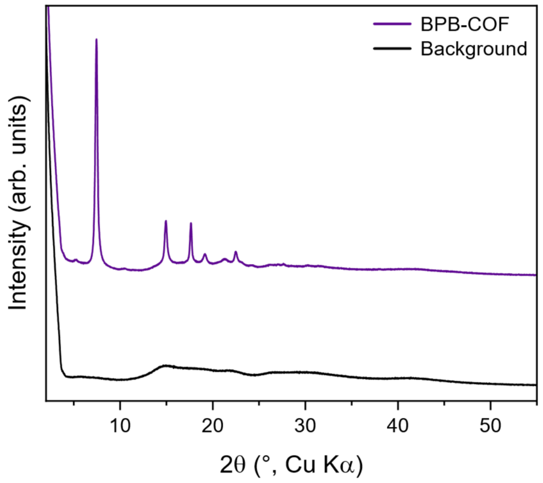

Supplementary Fig. 2: PXRD pattern of BPB-COF obtained after 12 days of solvothermal treatment (without background subtraction) and blank measurement with background resulting from Kapton foil and specimen holder.

4. Calculation of Interpenetration Degree

According to previous reports[1] the degree of interpenetration for diamond-net based frameworks either by $N = \frac{2\sqrt{4L^2-2a^2}}{c}$ (for *P*-lattice structures with *N* as even number) or $N = \frac{2\sqrt{4L^2-a^2}}{c}$ (for *I*-lattice structures where *N* is an odd number) with *N* being the degree of interpenetration, *L* the linker length and *a* and *c* being the lattice constants. For the linker length two borate nodes connected by 4,4'biphenyl and the chain terminated by using 3 phenol ligands for each borate site were simulated and geometry optimized using the *Forcite* module withing *Materials Studio*, resulting in a respective node-to-node distance of 11.7 Å. With lattice constants of *a* = 16.7557 Å and *c* = 5.0126 Å the following degrees of interpenetrations were obtained:

P-lattice: Not solvable (negative number)

I-Lattice: 6.5-fold interpenetration

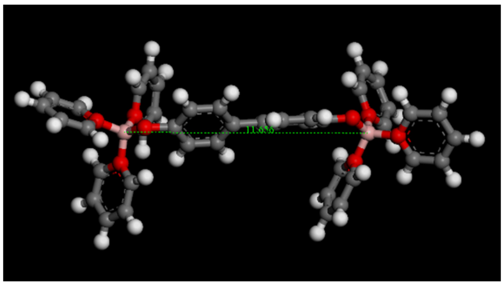

Supplementary Fig. 3: Geometry optimized model for the determination of the node-to-node distance.

5. Simulation and Refinement Data

For the structure simulation of **BPB-COF**, the experimental powder diffraction pattern was first indexed on a primitive tetragonal unit cell (*P*-lattice) with lattice constants of *a* = b = 16.7557 Å and *c* = 5.0126 Å (TREOR[2], figures of merit = 24). Due to the combination of tetrahedral $[BO_4]^-$ sites with ditopic linkers we assumed the formation of a regular 3D net with diamond topology BPB-COF should crystallize in a 3D framework formed of a single (dia) or, most likely, intergrown diamond net (dia-c*N*, with *N* defined as the number of interpenetrating net components).[3] To deduce the interpenetration degree *N* of BPB-COF, we tried using a general formula established in a previous study which is used for dia-based COF structures.[1] As no sensible result could be obtained utilizing this method, we started to investigate other possible topologies. The *PowderSolve*[4] algorithm within the *Reflex* module of *Materials Studio* was used to perform Monte Carlo parallel tempering to search for possible arrangements and conformations for the biphenyl linker within the cell. A good fit was found for a sql-net based model which was simulated expanding on these findings. As the *PowderSolve*[4] algorithm disregards energetic components of the structure, we then performed Pareto optimization to calculate a set of possible optimal refinement solutions in a series of Rietveld refinements in conjunction with the potential energy of the structure. Here, the *Universal force field*[5] is applied and a combined figure of merit (R_{comb}) is optimized, not only matching the experimental diffraction data (R_{wp}) but also minimizing the potential energy of the simulated structure (R_e). Different mixing factors are used, varying the emphasis placed on the potential energy and pattern fitting. All obtained results are in close agreement and we decided for a solution with a mixing factor of 75% with a R_{comb} of 1.72%, putting emphasis on the potential energy of the structure over the R_{wp} value. The final obtained structure was used for a final Rietveld refinement[6] with fixed structural parameters not varying the potential energy of the structure resulting in good final R_{wp} and R_p values of 6.51% and 3.39% and sensible bond distances of O–Li = 1.978 Å and O–B = 1.493.[7,8] (Final cell parameters; P-4, No.81, *a* = b = 16.72881 Å and *c* = 5.01643 Å, Boron (Wyckoff 1b & 1d), Lithium (Wyckoff 1a & 1c) Fig. 3).

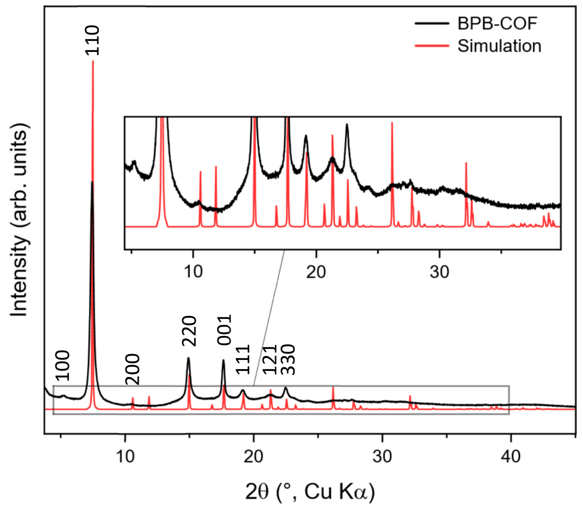

Supplementary Fig. 4: Comparison of the experimental PXRD pattern of BPB-COF (12 days) and calculated pattern based on the simulated ***sql****-net with inset highlighting low intensity diffractions and HKL values.*

For the structure solution various models based on different topologies were generated. A square lattice (sql) was shown to be in agreement with the experimental powder diffraction data. As comparison dia-C2, dia-C3, dia-C4 and dia-C6 is shown.

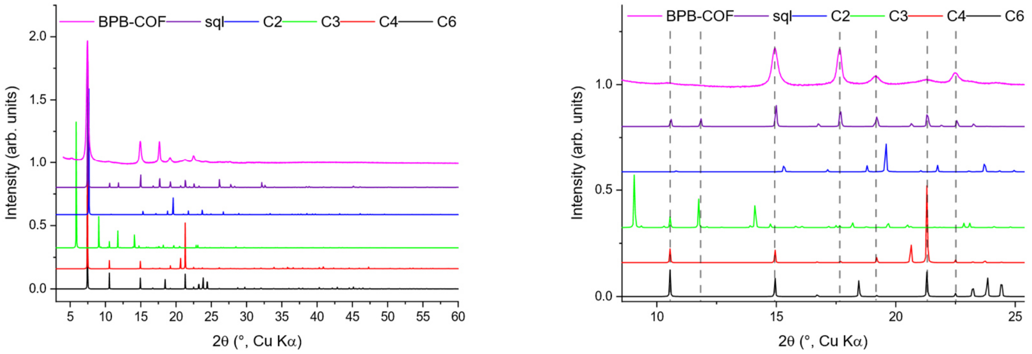

Supplementary Fig. 5: Calculated PXRD pattern for potential structural models.

6. Stability of BPB-COF

The stability of BPB-COF was evaluated by measuring an old sample of BPB-COF (12 days) which was stored in the glovebox for 3 months prior. The same PXRD sample was remeasured after being exposed to air.

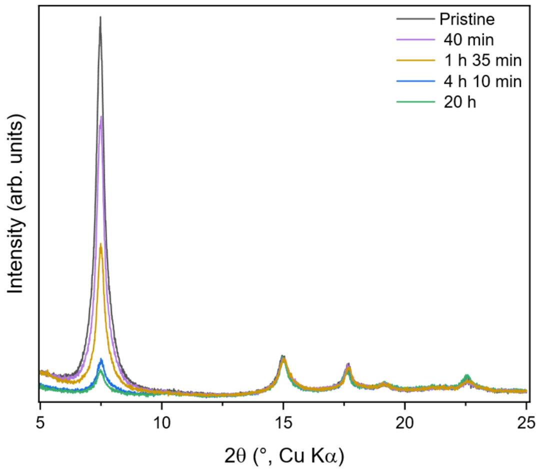

Supplementary Fig. 6: PXRD pattern of BPB-COF pristine and exposed to air (background subtracted).

To verify the thermal stability of BPB-COF (5 days) the compound was measured before and after sorption measurements. After 5 days of solvothermal synthesis the material was activated as described according to the procedure at 100 °C under vacuum. Additionally, before the sorption measurements the material was activated for 12 h at 150 °C under vacuum (10^{-3} mbar). No indication of decomposition in the PXRD can be seen showing the thermal stability of BPB-COF.

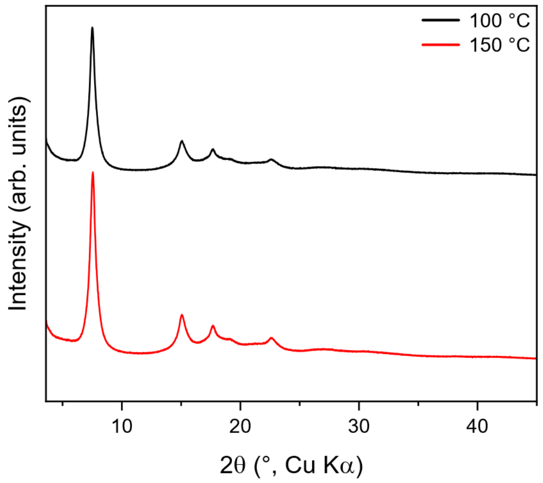

Supplementary Fig. 7: Before and after sorption measurements collected PXRD patterns of BPB-COF without background subtraction.

The ability of BPB-COF to retain its structural integrity was tested in dry solvents by suspending the powder under inert atmosphere and left standing over night before filtering and drying at 100 °C.

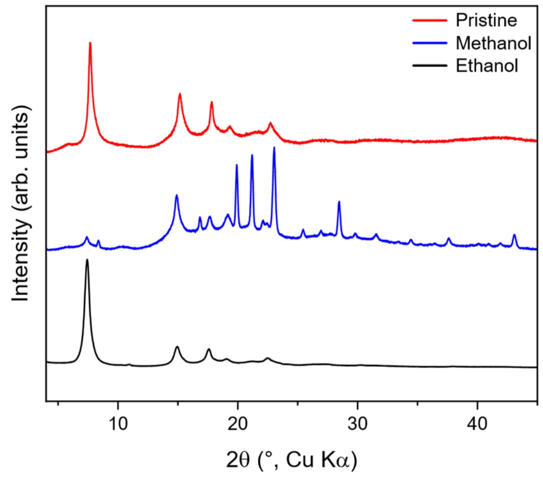

Supplementary Fig. 8: Stability of BPB-COF under methanol and ethanol.

7. Thermogravimetric Analysis (TGA)

Thermogravimetric analysis was performed under nitrogen atmosphere. The initial increase in weight can be attributed to the uptake of atmospheric moisture by the materials as they were for a short duration exposed to air when transferred into the device. Compared to the amorphous material the crystalline BPB-COF shows less initial uptake of moisture and weight loss.

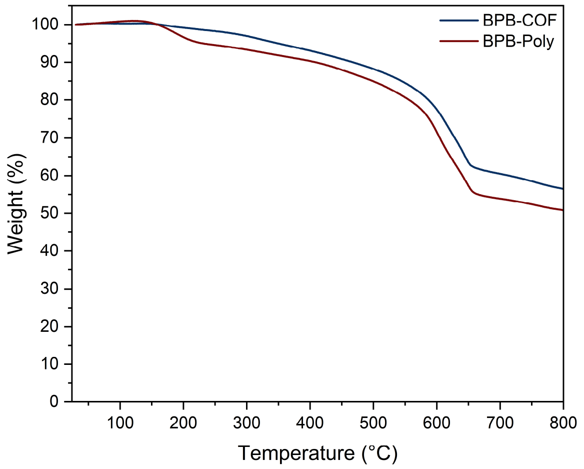

Supplementary Fig. 9: TGA of BPB-Poly and BPB-COF.

8. Sorption Measurements

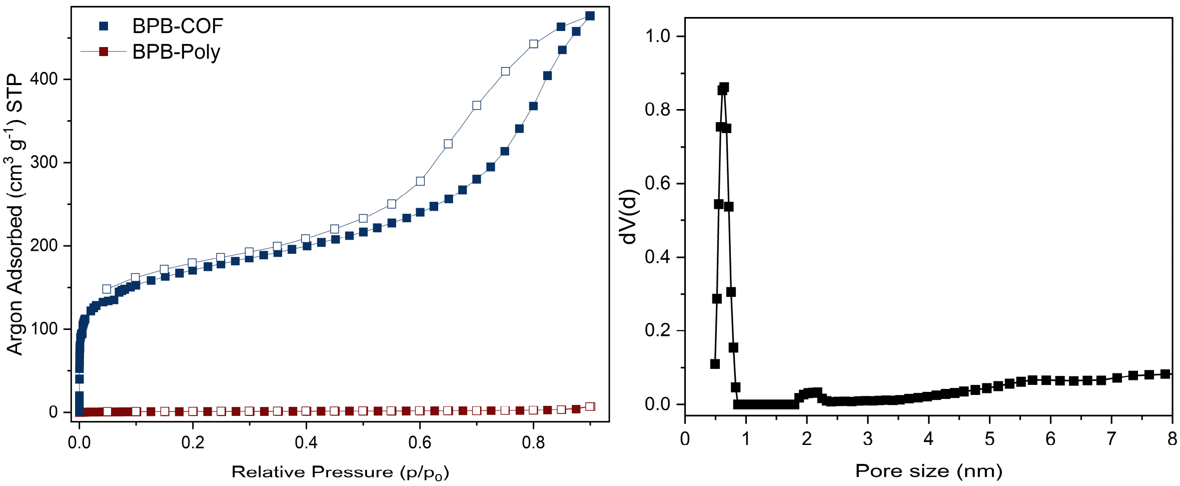

Supplementary Fig. 10: Argon 87K sorption isotherms for BPB-COF (5 days) and BPB-Poly (left) and pore size distribution (right).

9. BETSI Analysis

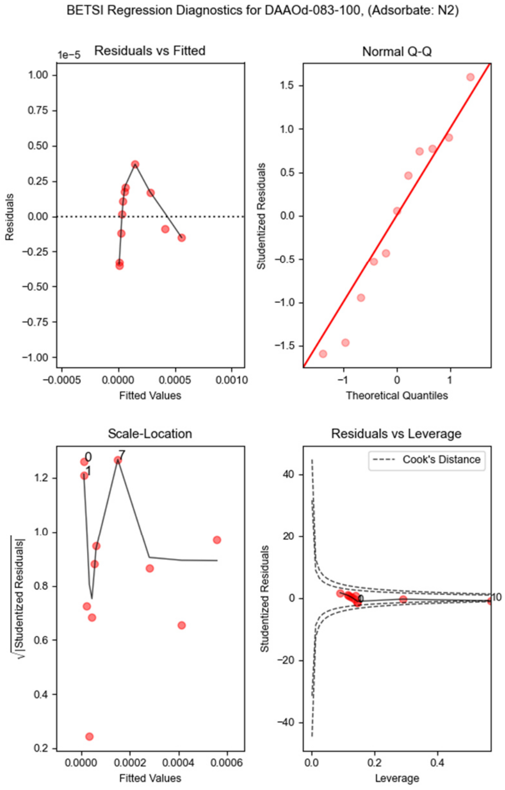

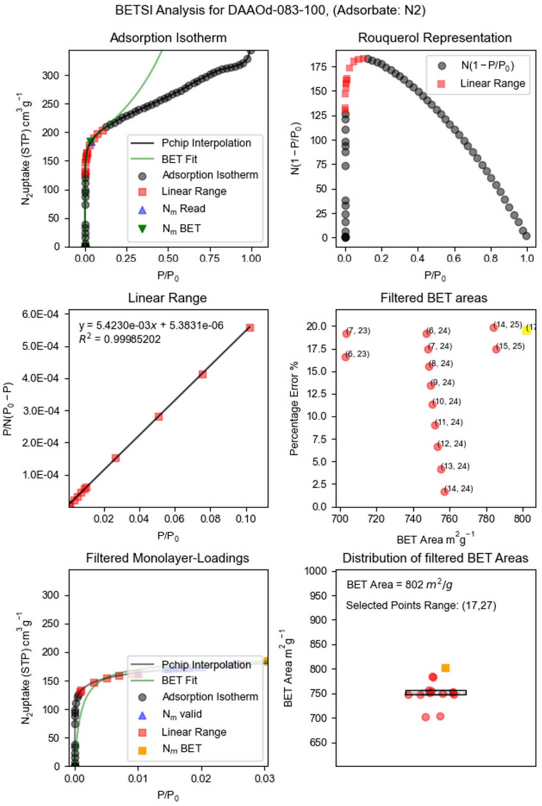

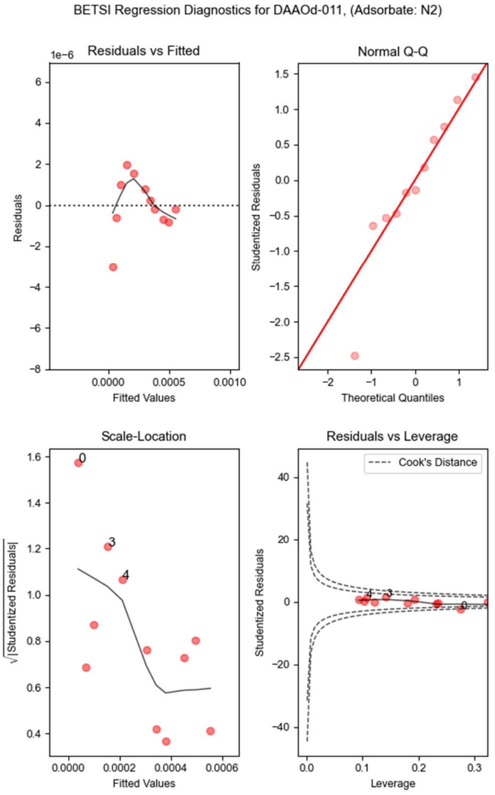

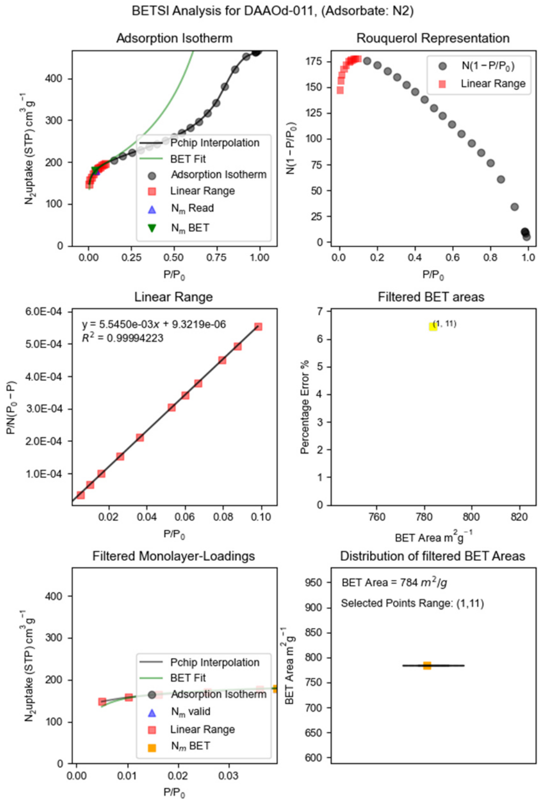

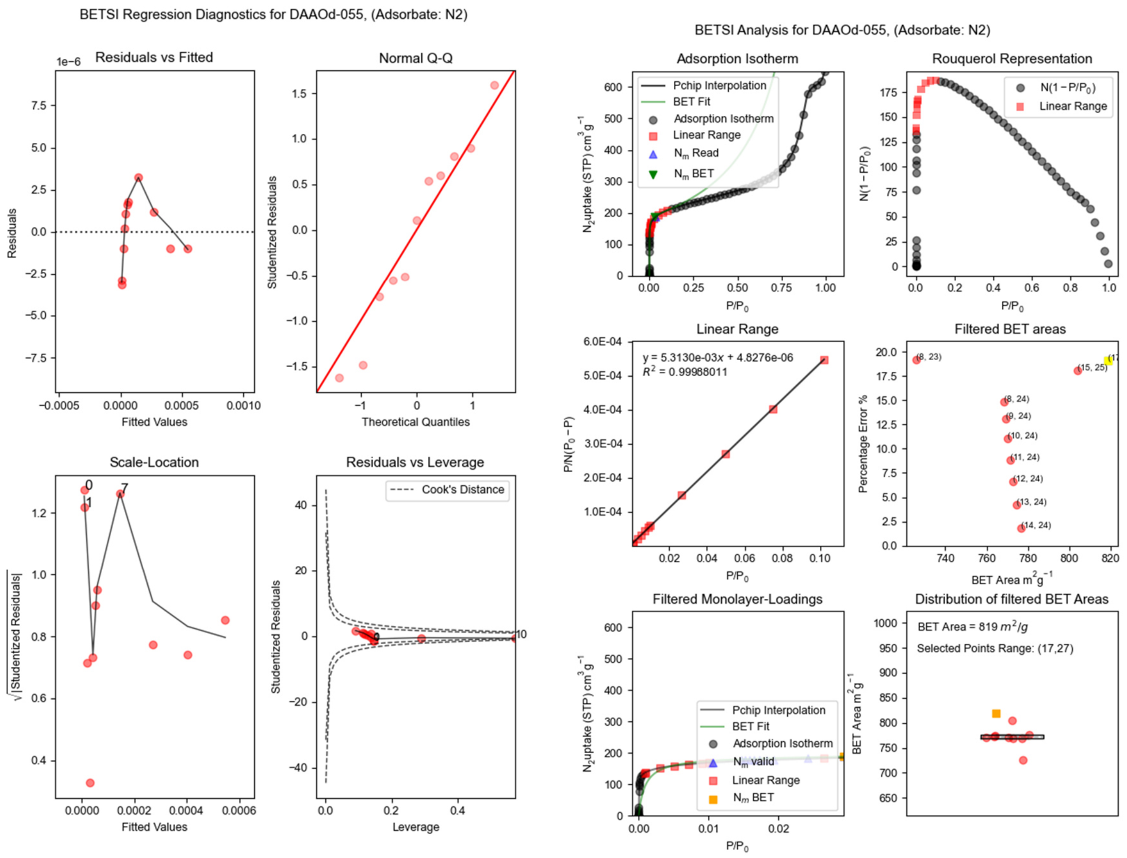

Supplementary Fig. 11: Calculation of surface areas using the BETSI tool.

10. Transmission Electron Microscopy (TEM)

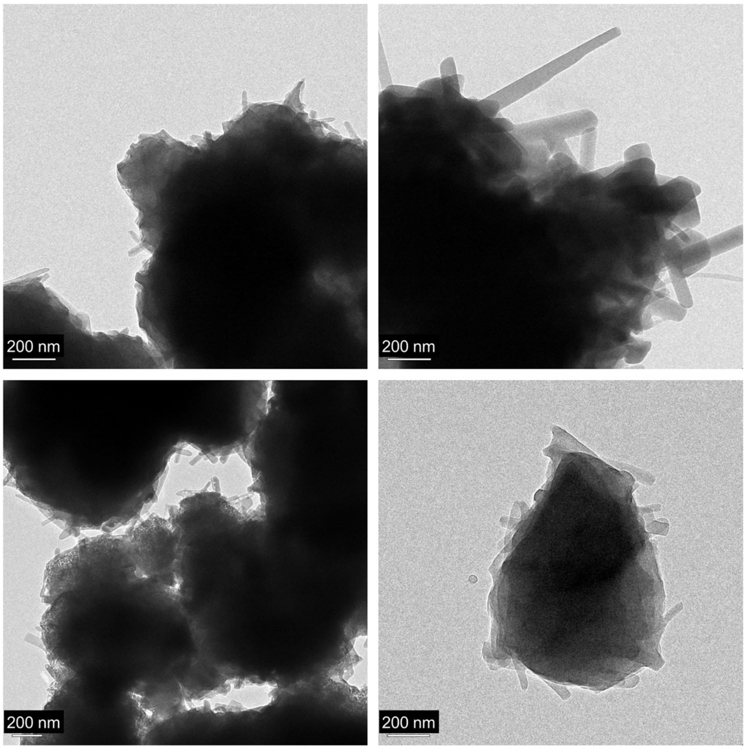

Supplementary Fig. 12: TEM images of BPB-Poly.

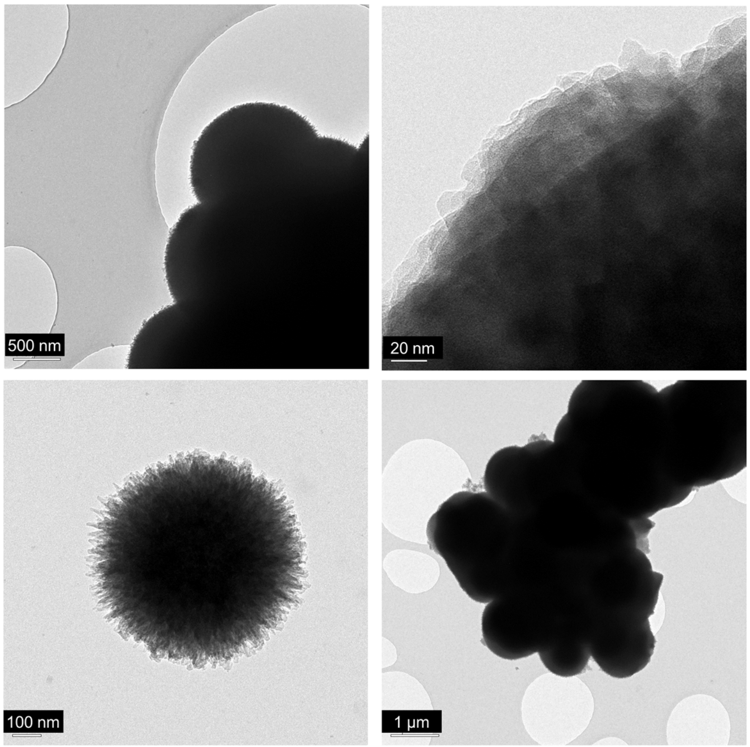

Supplementary Fig. 13: TEM images of BPB-COF after 2 days of solvothermal treatment.

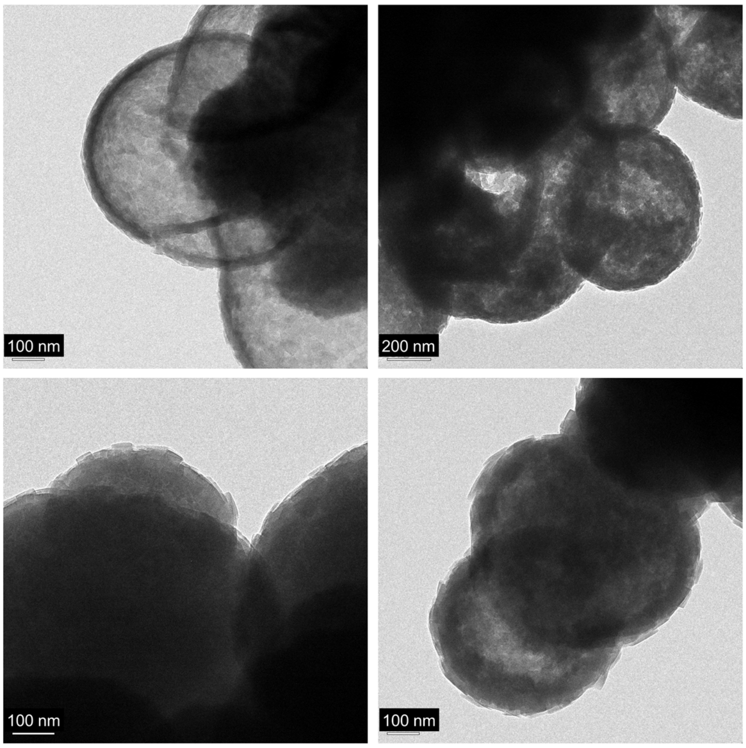

Supplementary Fig. 14: TEM images of BPB-COF after 5 days of solvothermal treatment.

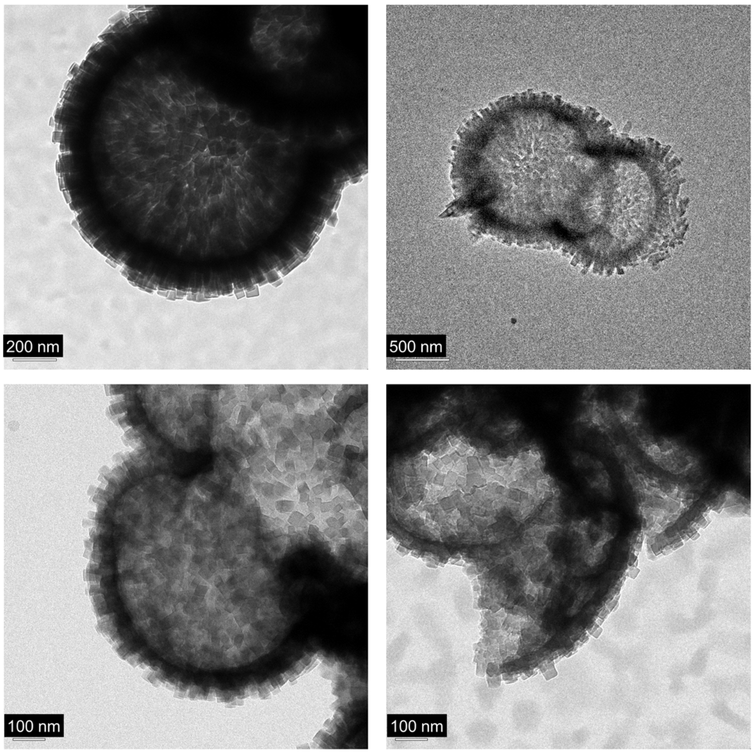

Supplementary Fig. 15: TEM images of BPB-COF after 12 days of solvothermal treatment.

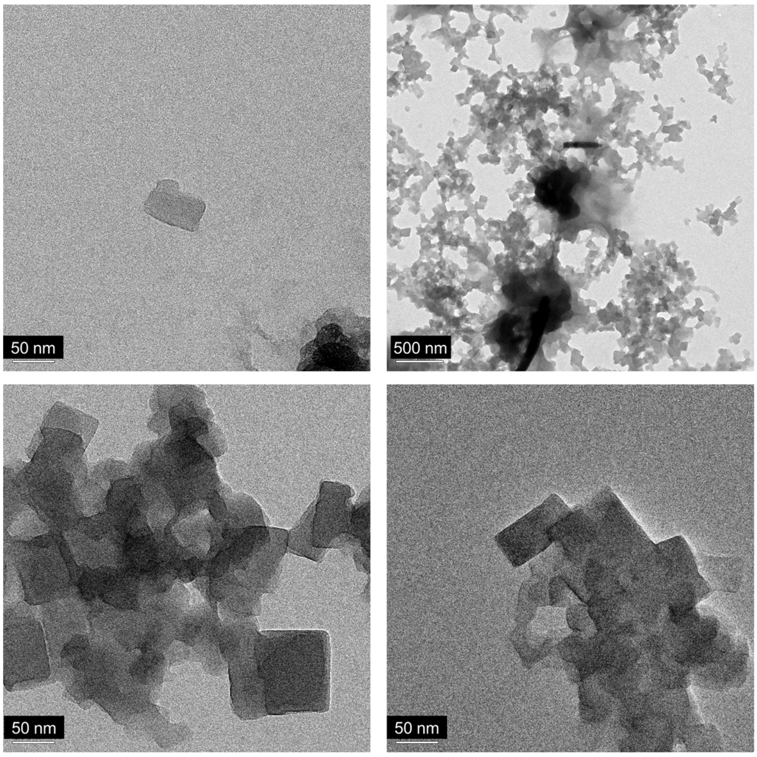

Supplementary Fig. 16: TEM images of the exfoliated BPB-NS.

11. Scanning Electron Microscopy (SEM)

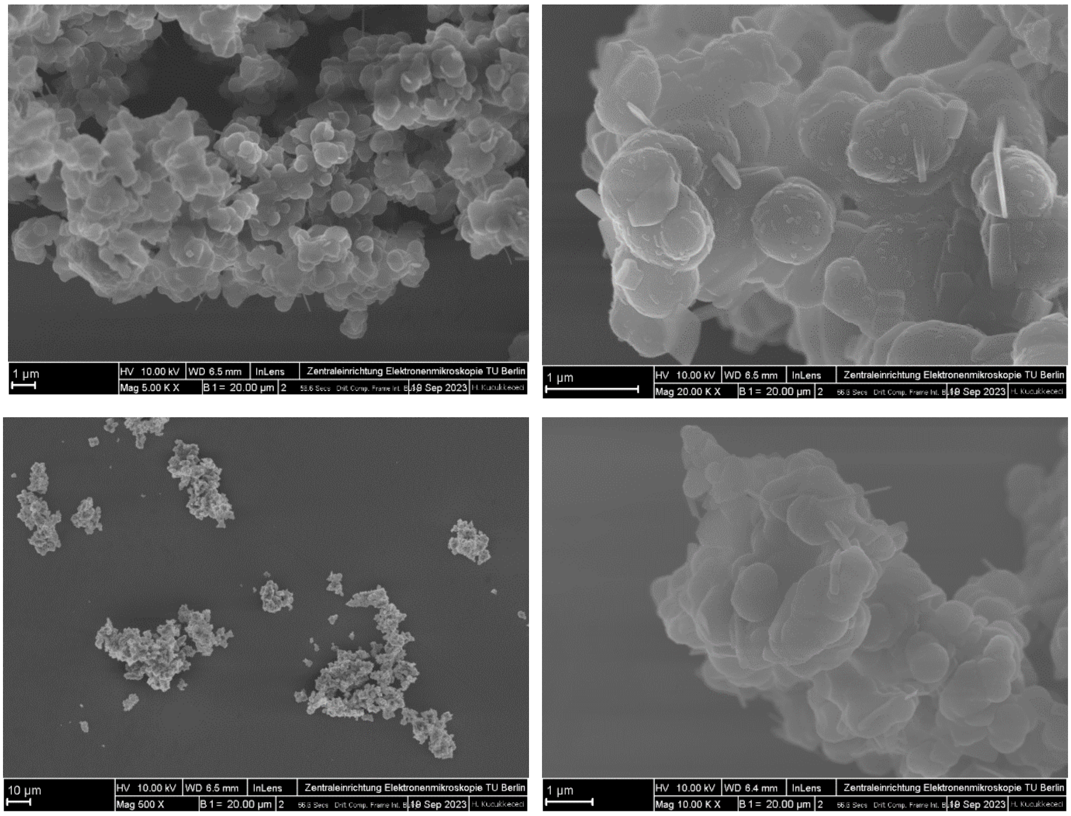

Supplementary Fig. 17: SEM images of BPB-Poly.

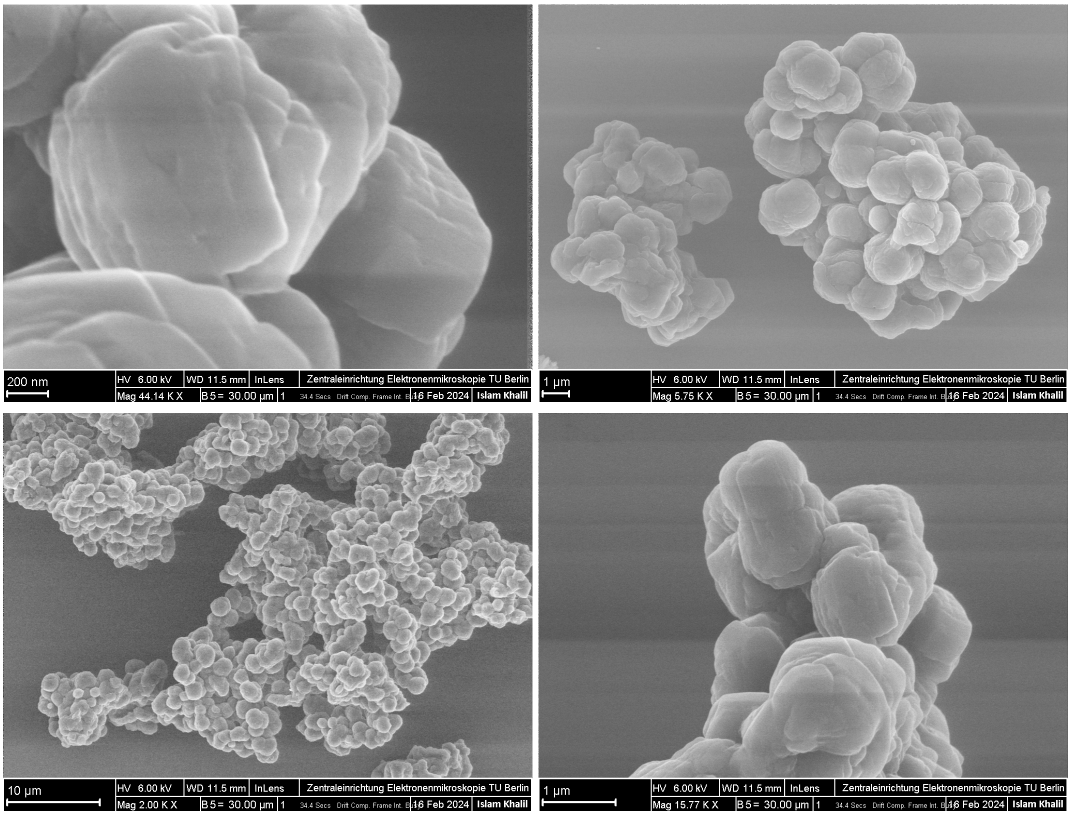

Supplementary Fig. 18: SEM images of BPB-COF after 2 days of solvothermal treatment.

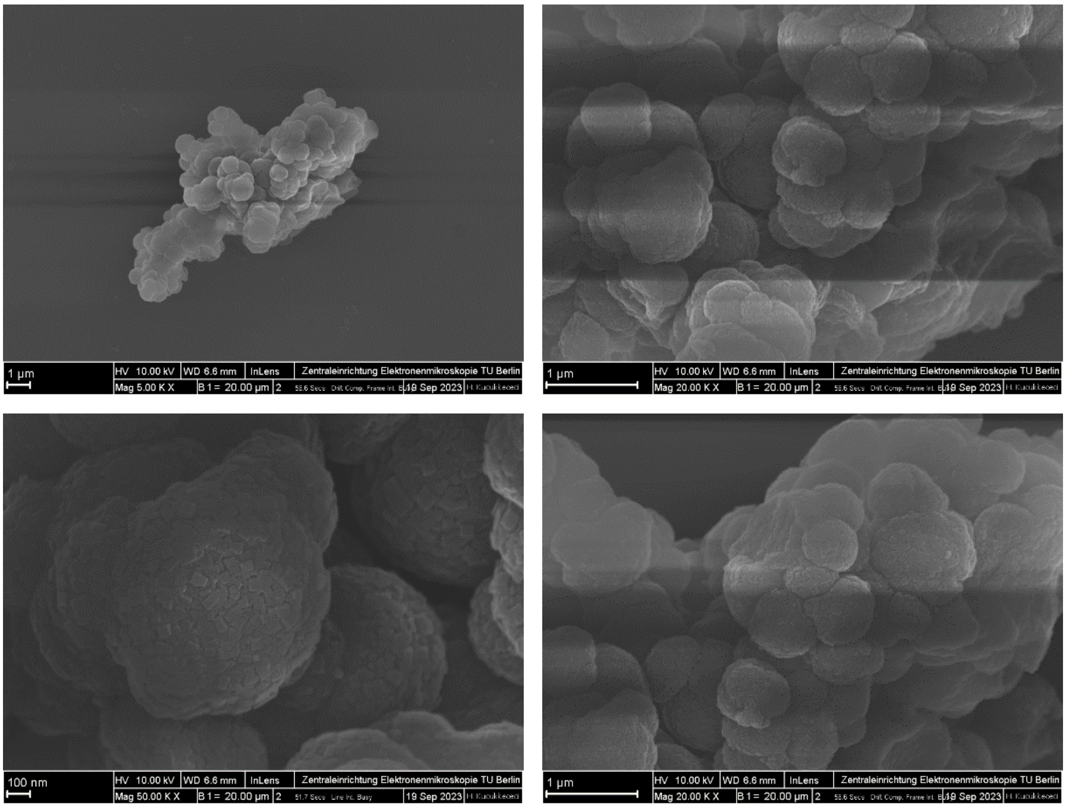

Supplementary Fig. 19: SEM images of BPB-COF after 5 days of solvothermal treatment.

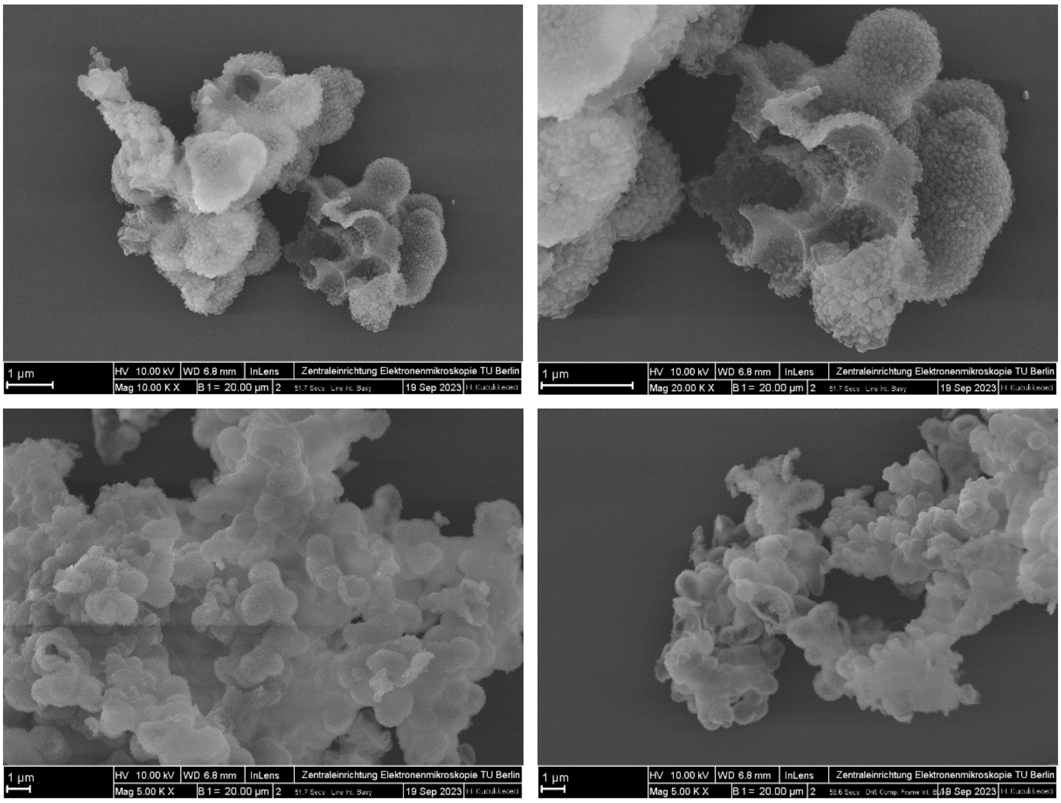

Supplementary Fig. 20: SEM image of BPB-COF after 12 days of solvothermal treatment.

12. Atomic Force Microscopy (AFM)

The roughness average (Ra) and root mean square of roughness (Rq) was determined through the height profile of a sample free space across the substrate visible as the vertical line (Supplementary Fig. 21b). For a larger sample size (Supplementary Fig. 21c) a mean height of 766 ± 8 pm was determined with heights primiarily <1 nm.

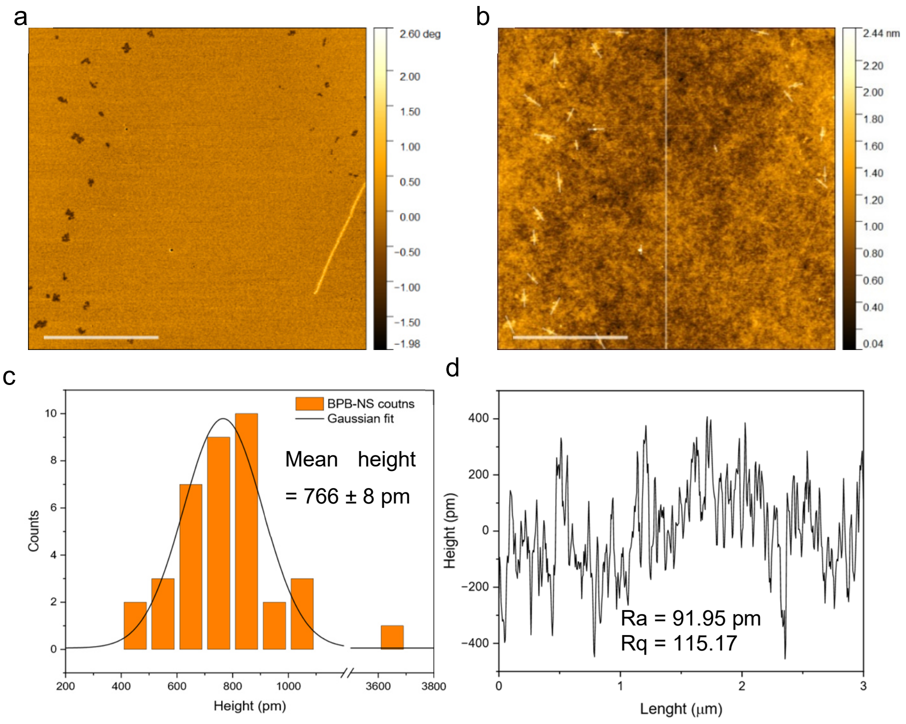

Supplementary Fig. 21: a) Phase image of BPB-NS on SiO2 substrate, scale bar is 400 nm. b) Height image of BPB-NS, scale bar is 400 nm. c) Histogram of in b) visible BPB-NS and Gaussian fit for the calculation of the mean height. d) Height profile across the substrate.

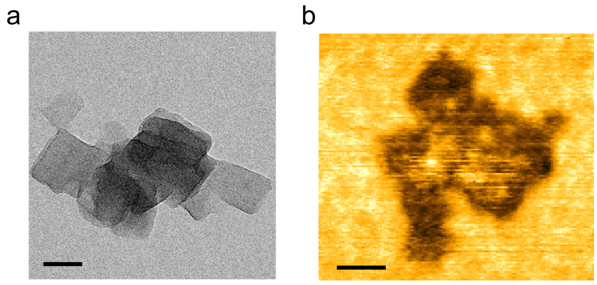

Supplementary Fig. 22: Side-by-side comparison of a) TEM and b) AFM roughness image of BPB-NS. Scale bars set to 50 nm.

13. Electron Impedance Spectroscopy (EIS)

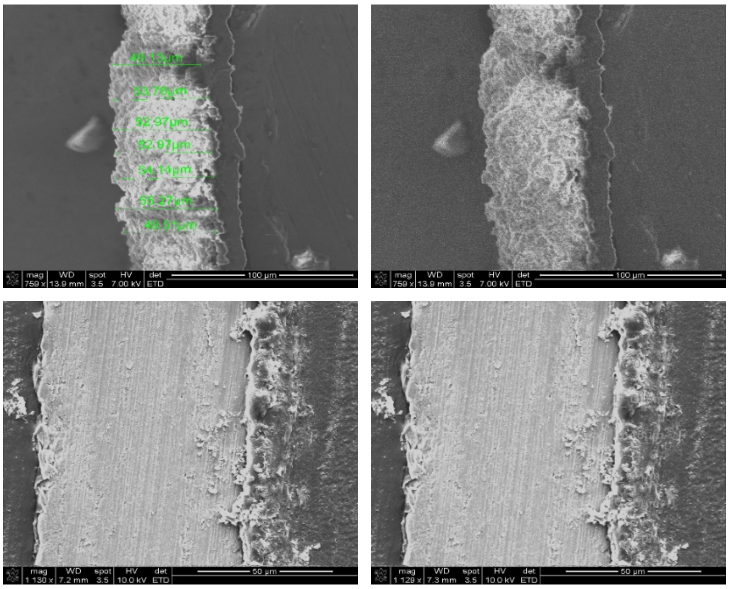

Supplementary Fig. 23: SEM image and measurement of the used for the determination of the average thickness of the electrode.

14. ICP-OES Measurements

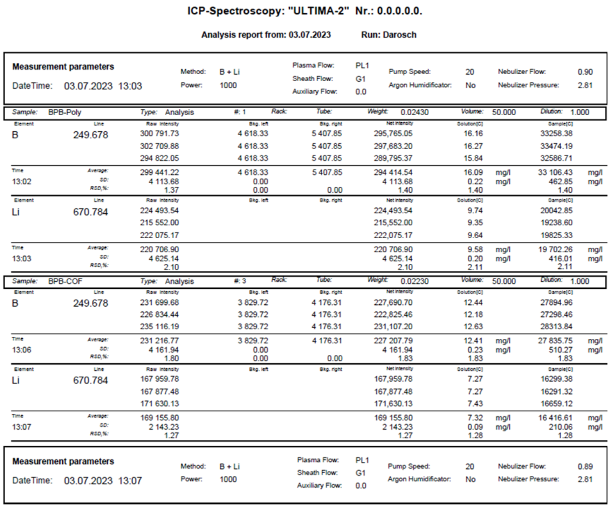

ICP-Spectroscopy: "ULTIMA-2" Nr.: 0.0.0.0.0.

Analysis report from: 03.07.2023 Run: Darosch

Measurement parameters — DateTime: 03.07.2023 13:03; Method: B + Li; Power: 1000; Plasma Flow: PL1; Sheath Flow: G1; Auxiliary Flow: 0.0; Pump Speed: 20; Argon Humidificator: No; Nebulizer Flow: 0.90; Nebulizer Pressure: 2.81

Sample: BPB-Poly; *Type:* Analysis; *#:* 1; *Rack:*; *Tube:*; *Weight:* 0.02430; *Volume:* 50.000; *Dilution:* 1.000

Element	Line	Raw Intensity	Bkg. left	Bkg. right	Net Intensity	Solution[C]		Sample[C]	
B	249.678	300 791.73	4 618.33	5 407.85	295,765.05	16.16		33258.38	
		302 709.88	4 618.33	5 407.85	297,683.20	16.27		33474.19	
		294 822.05	4 618.33	5 407.85	289,795.37	15.84		32586.71	
Time 13:02	Average:	299 441.22	4 618.33	5 407.85	294 414.54	16.09	mg/l	33 106.43	mg/l
	SD:	4 113.68	0.00		4 113.68	0.22	mg/l	462.85	mg/l
	RSD,%:	1.37	0.00	0.00	1.40	1.40		1.40	
Li	670.784	224 493.54			224,493.54	9.74		20042.85	
		215 552.00			215,552.00	9.35		19238.60	
		222 075.17			222,075.17	9.64		19825.33	
Time 13:03	Average:	220 706.90			220 706.90	9.58	mg/l	19 702.26	mg/l
	SD:	4 625.14			4 625.14	0.20	mg/l	416.01	mg/l
	RSD,%:	2.10			2.10	2.11		2.11	

Sample: BPB-COF; *Type:* Analysis; *#:* 3; *Rack:*; *Tube:*; *Weight:* 0.02230; *Volume:* 50.000; *Dilution:* 1.000

Element	Line	Raw Intensity	Bkg. left	Bkg. right	Net Intensity	Solution[C]		Sample[C]	
B	249.678	231 699.68	3 829.72	4 176.31	227,690.70	12.44		27894.96	
		226 834.44	3 829.72	4 176.31	222,825.46	12.18		27298.46	
		235 116.19	3 829.72	4 176.31	231,107.20	12.63		28313.84	
Time 13:06	Average:	231 216.77	3 829.72	4 176.31	227 207.79	12.41	mg/l	27 835.75	mg/l
	SD:	4 161.94	0.00		4 161.94	0.23	mg/l	510.27	mg/l
	RSD,%:	1.80	0.00	0.00	1.83	1.83		1.83	
Li	670.784	167 959.78			167,959.78	7.27		16299.38	
		167 877.48			167,877.48	7.27		16291.32	
		171 630.13			171,630.13	7.43		16659.12	
Time 13:07	Average:	169 155.80			169 155.80	7.32	mg/l	16 416.61	mg/l
	SD:	2 143.23			2 143.23	0.09	mg/l	210.06	mg/l
	RSD,%:	1.27			1.27	1.28		1.28	

Measurement parameters — DateTime: 03.07.2023 13:07; Method: B + Li; Power: 1000; Plasma Flow: PL1; Sheath Flow: G1; Auxiliary Flow: 0.0; Pump Speed: 20; Argon Humidificator: No; Nebulizer Flow: 0.89; Nebulizer Pressure: 2.81

Supplementary Fig. 24: Experimentally determined boron and lithium contents.

Atom	***%B***	***%Li***
**Calculated**	2.80	*1.80*
**BPB-Poly**	3.30±0.16	1.97±0.08
**BPB-COF**	2.78±0.12	1.64±0.07

Supplementary Table 2: Calculated and experimentally determined wt% of boron and lithium found for the amorphous and crystalline (5 days) polymer.

15. Theoretical Simulations of Chemical Shielding

The chemical shielding and electric field gradient tensors were calculated using the CASTEP module integrated within Materials Studio based on the CIF file of BPB-COF. The calculations were performed on Ultra-fine quality settings using GGA PBE as functional and OTFG ultrasoft for pseudopotential. To compare the calculated absolute chemical shielding with the experimental values Li_2O was used as reference point for the conversion of the calculated isotropic chemical shifts.[9]

$$\sigma_{iso}^{BPB-COF} - \sigma_{iso}^{Li_2O} = \mathit{\Delta}\,; 88.33\,ppm - 86.49\,ppm = 1.84\,ppm$$

$$\delta_{iso}^{Li_2O} - \mathit{\Delta} = \delta_{iso}^{BPB-COF}; 3.03\,ppm - 1.84\,ppm = 1.19\,ppm$$

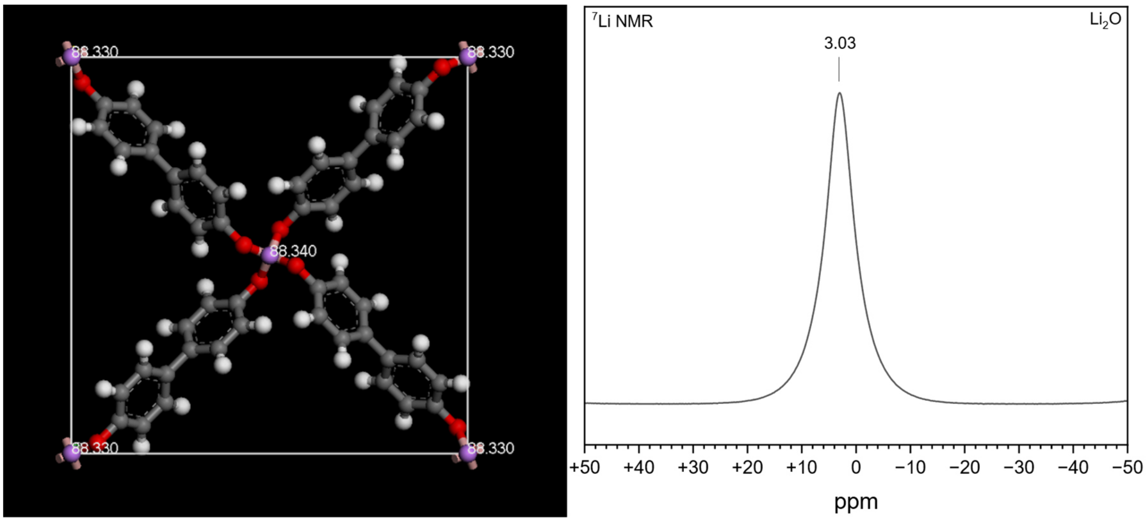

Supplementary Fig. 25: Unit cell of BPB-COF with the lithium ions labeled with the corresponding absolute isotropic chemical shift of 88.33/88.34 ppm (left) and Li_2O measured as reference.

16. Reflex Summary Report for Rietveld Refinement

Final R_{wp}:	6.51%	Final R_p:	3.39%
Final R_{wp} (without background):	24.96%	Final CMACS:	29.15%

Setup

2θ Range (degrees):	3.80-60.00	Step Size (degrees):	0.010
Experiment:	DAA0d-027-GB_Kapton_background_substracted_WAXS_exported_0_1.xcd		
Excluded Regions:	-		

Radiation

Type:	X-ray	Source:	Copper
λ (Å):	1.540.562	Monochromator:	None
Anom. Dispersion:	No	Polarization:	0.500

Lattice Parameters

Lattice Type:	Tetragonal	Space Group:	P -4

Parameter	Value	Refined?
a	16.72881 ± *0.00000*	Yes
b	16.72881 ± *0.00000*	Yes
c	5.01643 ± *0.00059*	Yes
α	9.000.000	No
β	9.000.000	No
γ	9.000.000	No

Structure Parameters

Refined Motion Groups:	0	Refined Torsions:	0
Refined Angles:	0	Refined Distances:	0
Number of Refined DOF:	0		

Fractional Coordinates

No.	Name	u	v	w	Refined?
1	C1	0.08298	0.87797	0.36788	No
2	C2	0.15295	0.87569	0.21626	No
3	C3	0.21100	0.81776	0.26782	No
4	C4	0.19965	0.76129	0.47242	No
5	C5	0.12891	0.76422	0.62379	No
6	C6	0.07101	0.82225	0.57145	No
7	C7	0.26127	0.69968	0.52766	No
8	C8	0.31779	0.71106	0.73177	No
9	C9	0.37572	0.65301	0.78332	No
10	C10	0.37794	0.58301	0.63219	No
11	C11	0.32217	0.57101	0.42910	No
12	C12	0.26413	0.62889	0.37682	No
13	O13	0.02626	0.93466	0.31694	No
14	O14	0.43464	0.52629	0.68306	No
15	H15	0.16228	0.91863	0.05786	No
16	H16	0.26420	0.81646	0.14528	No
17	H17	0.11880	0.72238	0.78491	No
18	H18	0.01695	0.82399	0.68969	No
19	H19	0.31655	0.76432	0.85383	No
20	H20	0.41873	0.66237	0.94123	No
21	H21	0.32387	0.51691	0.31132	No
22	H22	0.22224	0.61877	0.21606	No
23	B23	0.50000	0.50000	0.50000	No
24	B24	0.00000	0.00000	0.50000	No
25	Li25	0.00000	0.00000	0.00000	No
26	Li26	0.50000	0.50000	0.00000	No

Occupancies

No.	Name	Occupancy	Refined?
1	C1	100.000	No
2	C2	100.000	No
3	C3	100.000	No
4	C4	100.000	No
5	C5	100.000	No
6	C6	100.000	No
7	C7	100.000	No
8	C8	100.000	No
9	C9	100.000	No
10	C10	100.000	No
11	C11	100.000	No
12	C12	100.000	No
13	O13	100.000	No
14	O14	100.000	No
15	H15	100.000	No
16	H16	100.000	No
17	H17	100.000	No
18	H18	100.000	No
19	H19	100.000	No
20	H20	100.000	No
21	H21	100.000	No
22	H22	100.000	No
23	B23	100.000	No
24	B24	100.000	No
25	Li25	100.000	No
26	Li26	100.000	No

Pattern Parameters

Profile Function: Pseudo-Voigt

FWHM

Parameter	Value	Refined?
U	6.90228 ± *0.00000*	Yes
V	-1.62709 ± *0.00000*	Yes
W	0.09463 ± *0.00000*	Yes

Profile

Parameter	Value	Refined?
NA	0.72640 ± *0.02603*	Yes
NB	0.00456 ± *0.00294*	Yes

Line Shift

Instrument Geometry: Bragg-Brentano

Parameter	Value	Refined?
Zero Point	-0.02784 ± *0.00066*	Yes
Shift #1	0.00000	No
Shift #2	0.00000	No

Asymmetry

Correction: Rietveld 2θ Limit: 90.000

Parameter	Value	Refined?
P	-0.04462 ± *0.00183*	Yes

Sample Parameters

Preferred Orientation

Function: March-Dollase

Parameter	Value	Refined?
a^*	-0.00245 ± *0.12253*	Yes
b^*	-0.00230 ± *0.12325*	Yes
c^*	0.99999 ± *0.00041*	Yes
R0	0.81328 ± *0.00464*	Yes

Crystallite Size

Parameter	Value	Refined?
A	529.21086 ± *5.59921*	Yes
B	529.21086 ± *5.59921*	Yes
C	591.76509 ± *24.84886*	Yes

Lattice Strain

Parameter	Value	Refined?
A	0.01000	No
B	0.01000	No
C	0.01000	No

Global Isotropic Temperature Factors

Parameter	Value	Refined?
U	0.39591 ± *0.01201*	Yes

17. Supplementary References

(1) Ma, T.; Li, J.; Niu, J.; Zhang, L.; Etman, A. S.; Lin, C.; Shi, D.; Chen, P.; Li, L.-H.; Du, X.; Sun, J.; Wang, W. Observation of Interpenetration Isomerism in Covalent Organic Frameworks. *J. Am. Chem. Soc.* **2018**, *140* (22), 6763–6766. https://doi.org/10.1021/jacs.8b03169.

(2) de Wolff, P. M. A Simplified Criterion for the Reliability of a Powder Pattern Indexing. *J. Appl. Crystallogr.* **1968**, *1* (2), 108–113. https://doi.org/10.1107/S002188986800508X.

(3) O'Keeffe, M.; Peskov, M. A.; Ramsden, S. J.; Yaghi, O. M. The Reticular Chemistry Structure Resource (RCSR) Database of, and Symbols for, Crystal Nets. *Acc. Chem. Res.* **2008**, *41* (12), 1782–1789. https://doi.org/10.1021/ar800124u.

(4) Engel, G. E.; Wilke, S.; König, O.; Harris, K. D. M.; Leusen, F. J. J. PowderSolve – a Complete Package for Crystal Structure Solution from Powder Diffraction Patterns. *J. Appl. Crystallogr.* **1999**, *32* (6), 1169–1179. https://doi.org/10.1107/S0021889899009930.

(5) Rappe, A. K.; Casewit, C. J.; Colwell, K. S.; Goddard, W. A.; Skiff, W. M. UFF, a Full Periodic Table Force Field for Molecular Mechanics and Molecular Dynamics Simulations. *J. Am. Chem. Soc.* **1992**, *114* (25), 10024–10035. https://doi.org/10.1021/ja00051a040.

(6) Rietveld, H. M. A Profile Refinement Method for Nuclear and Magnetic Structures. *J. Appl. Crystallogr.* **1969**, *2* (2), 65–71. https://doi.org/10.1107/S0021889869006558.

(7) Wenger, M.; Armbruster, T. Crystal Chemistry of Lithium; Oxygen Coordination and Bonding. *Eur. J. Mineral.* **1991**, *3* (2), 387–399.

(8) Baur, W. H.; Fischer, R. X. The Floppiness of It All: Bond Lengths Change with Atomic Displacement Parameters and the Flexibility of Various Coordination Tetrahedra in Zeolitic Frameworks. An Empirical Structural Study of Bond Lengths and Angles. *Chem. Mater.* **2019**, *31* (7), 2401–2420. https://doi.org/10.1021/acs.chemmater.8b04919.

(9) Köcher, S. S.; Schleker, P. P. M.; Graf, M. F.; Eichel, R.-A.; Reuter, K.; Granwehr, J.; Scheurer, Ch. Chemical Shift Reference Scale for Li Solid State NMR Derived by First-Principles DFT Calculations. *J. Magn. Reson.* **2018**, *297*, 33–41. https://doi.org/10.1016/j.jmr.2018.10.003.
